# Self-assessment scale for the community-based and emergency practice

**DOI:** 10.1186/s12909-022-03848-z

**Published:** 2022-11-17

**Authors:** Takao Wakabayashi, Yoshihisa Tsuji, Takeshi Yamamoto, Hitoshi Sohma, Wari Yamamoto

**Affiliations:** 1 Department of General and Emergency Medicine, Japan Community Health-care Organization Sapporo Hokushin Hospital, Sapporo, Japan; 2 Department of General Medicine, Sapporo Medical University, S1 W17, Chuo-ku, Sapporo, 060-8556 Japan; 3 Department of Nursing, School of Health Sciences, Sapporo Medical University, S1 W17, Chuo-ku, Sapporo, 060-8556 Japan; 4 Center for Medical Education, Sapporo Medical University, S1 W17, Chuo-ku, Sapporo, 060-8556 Japan

**Keywords:** Clinical clerkship, Community-based, Emergency practice, Primary-care, Self-assessment

## Abstract

**Background:**

For current medical education, community-based primary care for the elderly is an essential topic. This study aimed to establish a scale of community-based assessment for clinical and emergency practice (C-CEP).

**Methods:**

A self-assessment scale for C-CEP was developed according to four steps. Initially, we reviewed publications from the societies of the United States, British, and Japan regarding educational goals. In addition, we searched MEDLINE for educational goals regarding attitude, skills, and knowledge. Getting together, we established 23 items as the educational goals of the C-CEP. Second, we collected responses for these 23 items from 5th-grade medical students (*n* = 195). Third, we conducted an exploratory factor analysis (EFA) using their responses to determine the fundamental structure of the self-assessment scale. Finally, a confirmatory factor analysis (CFA) was performed to assess the fitness of the self-assessment scale developing the EFA, resulting in modification of the items.

**Results:**

In EFA and CFA results, C-CEP Scale consisted of four factors with 15 items: “Attitude and communication in emergency care,” Basic clinical skills,” “Knowledge of community healthcare,“ and “Knowledge of evidence-based medicine perseverance.” The model fit indices were acceptable (Goodness of Fix Index = 0.928, Adjusted Goodness of Fit Index = 0.900, Comparative Fit Index = 0.979, and Root Mean Square Error of Approximation = 0.045). The values of McDonald’s omega as an estimate of scale reliability were more than 0.7 in all four factors. As for test-retest reliability, the intraclass correlation coefficients were ≥ 0.58 for all factors. All four factors of the C-CEP Scale correlated positively with the Medical Professionalism Evaluation Scale subscales.

**Conclusions:**

We developed a valid and reliable self-assessment scale to assess student competence.

**Supplementary Information:**

The online version contains supplementary material available at 10.1186/s12909-022-03848-z.

## Introduction

The world’s aging rate (aged 65 and older) has increased from 5.1% in 1950 to 9.0% in 2020. The United States (US) rate is estimated to elevate to 20% by 2040. For Japan, in 2020 had been about 20% already, but in many remote Japanese areas, the rate is over 40%.

Medicine’s goals in areas with many elderly residents are broad, diverse, and complex. Current outcomes of medical education in primary care are often designed based on these goals. However, it is pointed out that education designed from such complex outcomes can often increase educators’ burdens. One reason for the increasing burden is that the supervising doctor must teach medical students and residents in remote areas while caring for patients as a primary physician. It is not rare that medical resources, including human, in such rural area is limited.

For this reason, it appears that an efficient tool to educate the learner on primary care for the elderly in the rural community is essential [1–5]. Importantly, as per World Health Organization recommendations [6], primary care physicians who can care for patients comprehensively are also required. To educate medical students on attitudes, skills, and knowledge in the community [7], educators must prepare for community-based medical education (CBME) programs.

Although the effectiveness of the CBME program in training medical students and residents has been shown [8–34], no tool for self-assessment of the competencies in the CBME has been fully developed. For example, small studies using focus group interviews to assess these competencies were published. However, these methods were not simple and might increase the educator’s burden. There is a possibility that preparing enough opportunities to assess medical students by the instructor is difficult in rural areas due to work overload. A well-designed self-assessment method is required in CBME [35–42].

From this point of view, in this study, we aimed to develop a self-assessment scale for Community-based Clinical and Emergency Practice (C-CEP) and to verify its reliability and validity.

## Methods

### Study design

This study aimed to develop the C-CEP self-assessment scale according to these four steps. Initially, we reviewed publications from the societies of the US, British, and Japan regarding educational goals. In addition, we searched MEDLINE for educational goals regarding attitude, skills, and knowledge. Getting together, we established 23 items as the educational goals of the C-CEP. Second, we collected responses for these 23 items from 5th-grade medical students. Third, we conducted an exploratory factor analysis (EFA) using their responses to determine the fundamental structure of the self-assessment scale. A confirmatory factor analysis (CFA) was performed to assess the fitness of the self-assessment scale developing the EFA, resulting in modifying items without changes in the factor structure. Then, we calculated the McDonald’s omega coefficient was calculated to confirm the scale’s internal reliability. Finally, we investigated the validity and reliability of the self-assessment form compared to another scale. The Ethics Committee approved this study protocol of Sapporo Medical University (SMU) (3-1-58).

### The items on the self-assessment scale

To make a C-CEP self-assessment scale, we selected the goals published by domestic academic societies of medical education. This was to reflect the diversifying world educational goals on this self-assessment scale. We reviewed publications from the societies of the US, British, and Japan regarding educational goals; the American Medical Association (AMA) and the Association of American Medical Colleges (AAMC) [43], the British Medical Association (BMA) [44] and the British General Medical Council (GMC) [45]), and the Japanese government, specifically the Ministry of Education, Culture, Sports, Science and Technology (MEXT) [46] and the Japanese government, specifically Ministry of Health, Labour and Welfare (MHLW) [47]) (Supplementary1-6). Moreover, we reviewed MEDLINE from 2008 to 2020 using the following six keywords: medical education, community-based medical education, community-oriented medical education, emergency medicine, medical education, and primary care. According to the MEDLINE search and the reviews of international educational goals shown above, we established 23 items as the educational goals of C-CEP.

The responses for these 23 items were categorized based on the 5-grade self-assessment (5; strongly agree, 4; relatively agree, 3; equivocal, 2; relatively disagree, and 1; strongly disagree) and were recorded according to each student.

### Surveillance for medical clinical clerkship students

We conducted a cross-sectional survey of clinical clerkship and 5th-grade medical students from SMU between 2015 and 2016. All of these students participated in a two-week community-based clinical medicine educational program. The students assessed themselves using the 23 items self-assessment form before the start of the clerkship program.

### Exploratory factor analysis for the fundamental constructs of the question items and confirmatory factor analysis for the goodness of fit

We used the minimum average partial test (MAP) and Bayesian Information Criterion (BIC) to determine the number of factors. Then, EFA using the principal factor method with Promax rotation, was performed to clarify the underlying structures of factors. We excluded items for which all factor loadings were < 0.3. When there was multiple factor loading > 0.4, or when factor loading was > 0.4 for some and > 0.3 for others, the items were eliminated sequentially. The EFA was conducted to determine the factor structure; when factor loadings for all four factors were less than 0.35, the items were eliminated sequentially, and the factor analysis was repeated. A factor was defined as having at least three items.

For the factors extracted based on EFA, we adopted the quadratic factor analysis model because of its clarity of interpretation. We hypothesized that the factors identified by EFA would explain the upper factor, “Community-based Clinical and Emergency Practice,” in students who participated in CBME program at SMU. A CFA using covariance structure analysis was performed to verify the construct validity of the created scale using responses from 5th-grade students. Based on the theoretical model, the Goodness of Fix Index (GFI) and Adjusted Goodness of Fit Index (AGFI) are used as the goodness-of-fit indices. The Comparative Fit Index (CFI) and Root Mean Square Error of Approximation (RMSEA) were used as the criterion comparison indices. Without changes in factor structure, items were deleted as appropriate to improve the model based on the results. Model refinement was completed when the model had the highest goodness of fit and met the criterion comparison index. After the model was refined, McDonald’s omega coefficient was calculated to check the internal consistency of the constructed scale.

### Validity and reliability of the self-assessment scale

Surveillance was conducted in 2016 with 4th-grade medical students at SMU to validate the reliability of the C-CEP scale. Surveillance for the students, who participated in 2 weeks of the C-CEP program, was performed before and after the program. First, the scores of factors extracted by CFA were obtained from the pre-and post-questionnaires. Second, inter-rater reliability with intraclass correlation coefficient (ICC) was assessed to assess test-retest reliability. To validate reliability (Supplement 7), we compared the scores from our C-CEP at the before-program to those from the Medical Professional Evaluation Scale (MPES) [48]. To compare results to C-CEP, the four factors of the MPES, “collaboration,” “providing safe, quality care,” “reflective practice,” and “interest in community health,” were selected. We analyzed correlations between scores from C-CEP scale and those from these four MPES factors.

### Ethics

Before the students completed the questionnaires, we explained that their grades and credits earned were not affected by whether or not they participated. The written informed consent, which stated that the survey was anonymous and voluntary, and all data were deleted after use in this research, was obtained. We numbered the questionnaires and linked the records when examining the test-retest reliability. After that, the results were anonymized.

### Statistical analysis

Descriptive statistics are presented as mean ± standard deviation (SD). A *p*-value of < 0.05 was deemed to indicate statistical significance. A certain amount of data is necessary to obtain reliable results in CFA [49]. Generally, the minimum number of subjects to ensure the stability of the variance-covariance matrix is 100, with 4 to 10 subjects per variable. We set the number of subjects per item at 7. Since our questionnaire consisted of 23 items, we aimed for a minimum of 161 participants. All statistical analyses were performed using SPSS Statistics (IBM SPSS Statistics for Windows, Version 22.0, IBM Corp., Armonk, NY) and R (version 4.2.1).

## Results

### The items on the self-assessment scale

The 23 items of C-CEP (Table 1) were fully matched to the medical students’ core curriculum goals and objectives for Japanese residents (MEXT and MHLW). Meanwhile, comparing these 23 items to the goals/objectives of medical students and residents in the US, 87.0% (20/23) of medical students’ goals and 100% of residents’ objectives were matched. Compared to the British, 100% of medical students’ goals and 21.7% (5/23) of residents’ objectives were matched.

**Table 1:** Self-assessment items on questionnaire and reference

#		Self-assessment items on questionnaire	Corresponding learning objectives (Supple1-6)					
			US [43]		British [44, 45]		Japan [46, 47]	
			Medical student	Graduates	Medical student	Graduates	Medical student	Graduates
1	Attitude	Communication to patient in ER [10, 12, 14, 16, 18, 22, 24, 28]	3	III	C, D	b	A-4	A1,2, B4, C1,3
2		Communication among medical team in ER [10, 12, 14, 16, 18, 22, 32]	3	III	C	b	A-5	A1,2, B4, C1,3
3		Communication to staffs of other hospitals [10, 12, 14, 16, 18, 32]	3	III	C	B	A-5, 7	B4-6, C1-4
4	Skills	Medical interviews [12, 18, 20–22, 25, 28]	4	II, III,,IV, V	A, D	A	A-2, 4	A1, B1-4, C1-4
5		Physical examinations [12, 18, 20–22, 25, 28]	4	II, IV, V	A	i	A-2,3, F-3	B2,3, C1-3
6		Planning for examinations in ER [16, 18, 20–22, 25, 28, 34]	4	II, IV, V	A		A-2,3, F-1	B1-3, C3
7		Explaining to patients, to obtain consent of examinations [18, 20–22]	4	II, IV, V	A	Ii	A-2,3	B1-3, C3
8		Explaining the methods and indications for life support procedures [18, 20–22]	2,4	II, IV, V	A	Iv	A-2,3	B1-3, C2,3
9		The methods and indications of basic procedures [18, 20–22]	2,4	II, IV, V	A	ii,iii,v	A-2,3, G-3	B1-3, C1-3
10		The meanings of vital signs in ER [18, 20–22, 34]	2,4	II, IV, V	A	i,ii	A-2,3, G-2	B1-3, C3
11	Knowledge	The role of expert support in ER [9, 10, 18, 22]	6	II, III, V	B, C		A-5,6,7, F-2	A2, B4,7, C1-3
12		Handling of official forms in ER [8, 18, 27]	6	VI	B		A-6,7, F-2	A1-4, B6,7
13		The rule of application, confidentiality and disclosure of medical records [18, 22, 25, 27]	1	I, VI	D	a	A-1,6,7, F-2	A1-3, B6,
14		The composition of medical team in the emergency room [9–11, 14, 16, 29, 31]	6	IV, VI	C	a	A-5, F-2	B5,7, C3
15		The coordination of the medical team in the emergency room [9–11, 14, 16, 29, 31]	6	III, IV, VI	C		A-5, F-2	B5,7, C3
16		The role of medical insurance in the emergency room [8, 22, 27, 29, 33]	6	VI	A, D	a	A-6,7, F-2	B6,7, C3
17		The role of the practical institution in the community [8, 11, 15, 16, 19, 22–24, 27, 29, 31–33]	1,6	I, IV, VI	B	c	A-1,7, F-2	A1-4, B7, C4
18		The role of a primary-care physician in the emergency setting [13, 17, 19, 20, 22]	6	VI	B		A-7, F-2	A1-4, B7, C3
19		The difference in primary physician‘s roles according to communities [11, 13, 17, 19, 20, 23, 26]	6	IV, VI	B	c	A-7, F-2	A1,2, B7, C4
20		The roles of the primary-care physician at the institution [11, 13, 17, 24, 26, 33]	6	IV, VI	B		A-7, F-2	A1,2, B7, C4
21		The characteristics of the community’s health problems [13–15, 19, 23, 27, 29, 30, 32]	6	VI	B	c	A-1,7, F-2	B7, C4
22		Referring to the evidence, to solve clinical problems [11, 12]	2	II	A		A-8,9, F-2, G-2	A4, B-8,9
23		Explaining the management for common diseases, using evidence [11, 12]	2	II	A		A-8,9, F-2	A4, B-8,9

### Surveillance for medical clinical clerkship students

A cross-sectional survey of 237 5th-grade medical students at SMU was conducted between 2015 and 2016. Of these 237 patients, 42 were excluded due to lack of data, and 195 were included in the analysis. The means and standard deviations for each item, together with the item-total correlation analysis, are shown in Table 2. None of the items did show ceiling and/or floor effects.

**Table 2:** The results of exploratory factor analysis for self-assessment in pre-clinical clerkship program

#	C-CEP	Developed Questionnaire Items	Factor Loadings			
			Factor 1	Factor 2	Factor 3	Factor 4
2	Attitude and communication in emergency care	Communication among medical team in ER	0.975	−0.086	−0.055	0.06
1		Communication to patient in ER	0.945	−0.048	− 0.025	0.028
3		Communication to staffs of other hospitals	0.881	0.023	0.046	−0.089
8	Basic Clinical Skills	Explaining the methods and indications for life support procedures	−0.058	0.969	0.069	−0.299
7		Explaining to patients, to obtain consent of examinations	−0.016	0.875	−0.297	0.175
9		The methods and indications of basic procedures	−0.07	0.859	−0.007	−0.016
10		The meanings of vital signs in ER	−0.086	0.685	0.087	0.085
6		Planning for examinations in ER	0.267	0.530	0.070	0.012
5		Physical examinations	0.171	0.464	0.171	0.010
20	Knowledge of Community healthcare	The roles of the primary-care physician at the institution	−0.08	0.041	0.816	0.007
21		The characteristics of the community’s health problems	−0.014	−0.044	0.801	0.01
19		The difference in primary physician’s roles according to communities	−0.007	−0.009	0.782	0.014
17		The role of the practical institution in the community	0.002	−0.151	0.745	0.092
18		The role of a primary-care physician in the emergency setting	0.065	−0.107	0.727	0.119
16		The role of medical insurance in the emergency room	0.044	0.195	0.565	−0.222
22	Knowledge of evidence-based medicine	Referring to the evidence, to solve clinical problems	0.004	−0.147	−0.055	0.813
23		Explaining the management for common diseases, using evidence	0.063	0.108	0.150	0.509
13		The rule of application, confidentiality and disclosure of medical records	−0.09	0.047	0.328	0.411
14		The composition of medical term in the emergency room	−0.021	0.209	0.177	0.382
11		The role of expert support in ER	0.075	0.307	0.139	0.379

### Exploratory factor analysis for the fundamental constructs of the question items and confirmatory factor analysis for the goodness of fit

MAP and BIC suggested three and six factors should be retained, respectively. Therefore, the four- and five-factors solutions were sequentially examined. According to EFA for self-assessment results from students who participated in the pre-clinical clerkship program (Table 2), items 4, 12, and 15 were eliminated. Thereby, we extracted four factors consisting of 20 items from the pre-analysis, and each factor had at least three items, and no factors were deleted. This resulted in a quadratic factor model consisting of four factors.

**Fig. 1:**
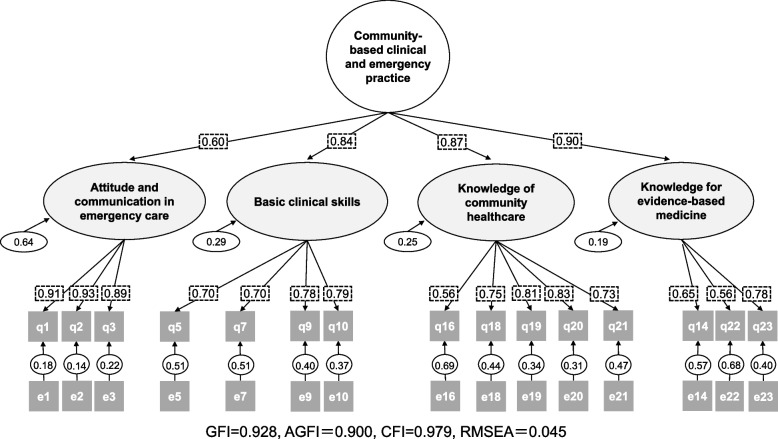
Path diagram of the C-CEP scale (after confirmatory factor analysis; CFA). The numbers surrounded by the dot-line in Fig. 1 represent standardized estimates. All values are *p* < 0.001. Abbreviation; e; error term, q; question item GFI; Goodness of fit index, AGFI; Adjusted goodness of fit index, RMSEA; Root mean square error of approximation

We then conducted a CFA on the 20-item model generated from the EFA, using the results of our 5th-grade students. However, among the goodness-of-fit indices, CFI was low at 0.927, and RMSEA was high at 0.073. Therefore, the goodness-of-fit indices were not met when 20 items were used as latent variables. After further analysis, items 6, 8, 11, 13, and 17 were eliminated. We extracted four factors comprising 15 items from the pre-analysis. The results were significant for all coefficients (standardized estimates) at the 5% level. In terms of goodness of fit indices, GFI = 0.928, AGFI = 0.900, CFI = 0.979, and RMSEA = 0.045, indicating a satisfactory fit. All factors showed good coefficients with the upper model (Fig. 1). McDonald’s omega coefficients were 0.933 for “Attitude and communication in emergency care” (3 items), 0.832 for “Basic clinical skills” (4 items), 0.864 for “ Knowledge of Community healthcare” (5 items), and 0.700 for “ Knowledge of evidence-based medicine” (3 items).

### Validation of the reliability of the self-assessment scale

Of the total 4th-grade medical students (*n* = 113), 79 were included in this study. Due to disagreement about participating in this study, 17 were excluded. According to the analysis of inter-rater reliability validation to assess test-retest reliability, “Attitude and communication in emergency care,” “Basic clinical skills,” and “ Knowledge of Community healthcare” showed substantial reliability; ICC (1,1) = 0.712, 0.659, 0.631, *p* < 0.001, respectively. Only “Knowledge of medical care regarding emergency room” showed moderate reliability; ICC (1.1) = 0.589, *p* < 0.001. Finally, we compared the factors of our scale to those of the MPES (Table 3). All of the factors developed correlated with each of the MPES items (*p* < 0.001). The high correlations (r > 0.70) were shown between “Knowledge of community healthcare” or “ Knowledge of evidence-based medicine” in C-CEP and “Interest in community health” in MEPS, “Basic clinical skills” in C-CEP and “providing safe, quality care” in MEPS, and “ Knowledge of evidence-based medicine” in C-CEP and “Interest in community health” in MEPS.

**Table 3:** Correlation between C-CEP Scale and MEPS

		Factors for C-CEP			
		Attitude and communication in emergency care	Basic clinical skills	Knowledge of community healthcare	Knowledge of evidence-based medicine
		*r*	*r*	*r*	*R*
Factors of MEPS	Collaborative practice	0.505*	0.588*	0.631*	0.584*
	Providing safe, quality care	0.636*	0.734*	0.635*	0.701*
	Reflective practice	0.455*	0.522*	0.436*	0.514*
	Interest in community health	0.583*	0.690*	0.733*	0.766*

*p < 0.001

*C-CEP Scale* self-assessment scale for the Community-based Clinical and Emergency Practice, *MEPS* Medical Professionalism Evaluation Scale

## Discussion

We developed a C-CEP scale as a novel tool for the self-assessment of community-based clinical practice. Using EFA and CFA, a 15-item questionnaire was developed to assess its internal reliability, retest reliability, and criterion-related validity. These results showed that students recognized the learning model of “Community-based Clinical and Emergency Practice” consisted of “Attitude and communication in emergency care,” “Basic clinical skills,” “Knowledge of Community healthcare,” and “Knowledge of evidence-based medicine” as essential competencies of their training. Since this scale is based on self-assessment with high validity and reliability, a reduced educator burden in remote medicine will be expected.

These four factors were considered to have certain reliability for self-assessment in community health care education, according to the results of CFA and comparisons with the MPES. In remote areas, educational resources are limited. In rural medicine education, self-learning tools are essential; e-learning and the Internet seem helpful. Notably, self-assessment experiences such as our study are helpful for the learners, who can train themselves to see a bird’s eye view, resulting in autonomous development. For students to acquire the attitude for growth in lifelong, it would be desirable to have the experience of self-assessment using a reliable device, such as our self-assessment scale.

MPES, comparable to C-CEP, is the self-assessment scale to assess the general capability of a medical doctor and professionalism. It consisted of 30 items from 7 factors for assessing students before clinical practice. The construct validity, criterion-related validity, and reliability of the MPES were generally confirmed and widely accepted. It is noteworthy that the C-CEP scale focuses on community-based emergency care capacity. This self-assessment scale correlated with the MPES because there is a partial overlap in the underlying competencies. We believe that using the two differently will have a higher educational effect.

This study has several limitations. Initially, the placements only lasted 2 weeks. Therefore, the readiness of motivation or fundamental knowledge of the learner might have affected the results. Second, the target group was also limited to a cohort of students at SMU, so it will be necessary to investigate whether the C-CEP Scale can be used in different cultures or languages. Third, the long outcome could not be assessed due to the short duration of the observation in this study. Finally, it is unclear if it can use this C-CEP when a pandemic like COVID-19 arises.

## Conclusion

The C-CEP Scale comprises 15 items that cover four factors and is both valid and reliable. This scale would help clerkship education and may also be used to improve its curricula.

## Supplementary Information

**Additional file 1: Supplementary 1.** Recommendations for Clinical Skills Curricula for Undergraduate Medical Education [43]**. Supplementary 2.** The duties of a doctor registered with the General Medical Council 2016, UK [44]. **Supplementary 3.** General Medical Council for graduates 2018 [45]. **Supplementary 4.** General Medical Council for graduates: Practical skills and procedures-practical 2019 [45]. **Supplementary 5.** Model Core Curriculum for Medical Education in Japan 2016 [46]. **Supplementary 6.** Basic qualities and abilities required of a physician 2020 [47]. **Supplementary 7.** Medical professional evaluation scale [48]

## Data Availability

The datasets used and/or analyzed during the current study are available from the corresponding author on reasonable request.

## Abbreviations

CBME: Community-based medical education
C-CEP: Community-based Clinical and Emergency Practice
EFA: Exploratory factor analysis
CFA: Confirmatory factor analysis
AMA: American Medical Association
AAMC: Association of American Medical Colleges
BMA: British Medical Association
GMC: General Medical Council
MEXT: Ministry of Education, Culture, Sports, Science and Technology
MHLW: Ministry of Health, Labour and Welfare
SMU: Sapporo Medical University
GFI: Goodness of Fix index
AGFI: Adjusted Goodness of Fit index
CFI: Comparative Fit Index
RMSEA: Root Mean Square Error of Approximation
ICC: Intraclass correlation coefficient
MPES: Medical Professional Evaluation Scale
SD: Standard deviation
