# Supplementary material for: Self-assessment scale for the community-based and emergency practice
Source: BMC Med Educ. 2022 Nov 17;22:799. doi: 10.1186/s12909-022-03848-z (PMC9670040; doi:10.1186/s12909-022-03848-z)
Supplement: Supplementary file 1 — Additional file 1: Supplementary 1. Recommendations for Clinical Skills Curricula for Undergraduate Medical Education [43]. Supplementary 2. The duties of a doctor registered with the General Medical Council 2016, UK [44]. Supplementary 3. General Medical Council for graduates 2018 [45]. Supplementary 4. General Medical Council for graduates: Practical skills and procedures-practical 2019 [45]. Supplementary 5. Model Core Curriculum for Medical Education in Japan 2016 [46]. Supplementary 6. Basic qualities and abilities required of a physician 2020 [47]. Supplementary 7. Medical professional evaluation scale [48] [file 12909_2022_3848_MOESM1_ESM.pptx]

## Slide 1
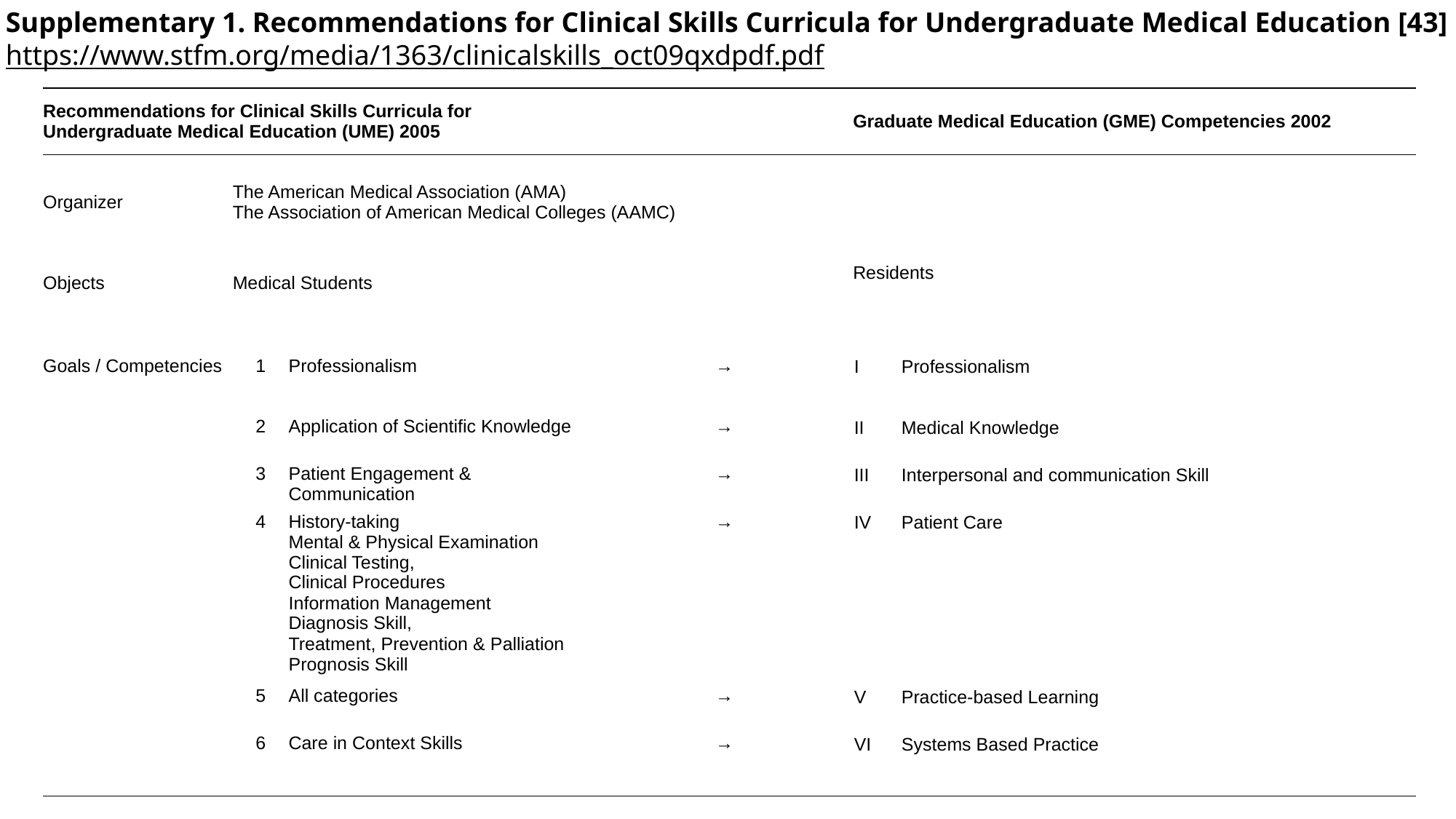

Supplementary 1. Recommendations for Clinical Skills Curricula for Undergraduate Medical Education [43]
https://www.stfm.org/media/1363/clinicalskills_oct09qxdpdf.pdf
| Recommendations for Clinical Skills Curricula for Undergraduate Medical Education (UME) 2005 | | | | Graduate Medical Education (GME) Competencies 2002 | |
| --- | --- | --- | --- | --- | --- |
| Organizer | The American Medical Association (AMA) The Association of American Medical Colleges (AAMC) | | | | |
| Objects | Medical Students | | | Residents | |
| Goals / Competencies | 1 | Professionalism | → | I | Professionalism |
| | 2 | Application of Scientific Knowledge | → | II | Medical Knowledge |
| | 3 | Patient Engagement & Communication | → | III | Interpersonal and communication Skill |
| | 4 | History-taking Mental & Physical Examination Clinical Testing, Clinical Procedures Information Management Diagnosis Skill, Treatment, Prevention & Palliation Prognosis Skill | → | IV | Patient Care |
| | 5 | All categories | → | V | Practice-based Learning |
| | 6 | Care in Context Skills | → | VI | Systems Based Practice |

## Slide 2
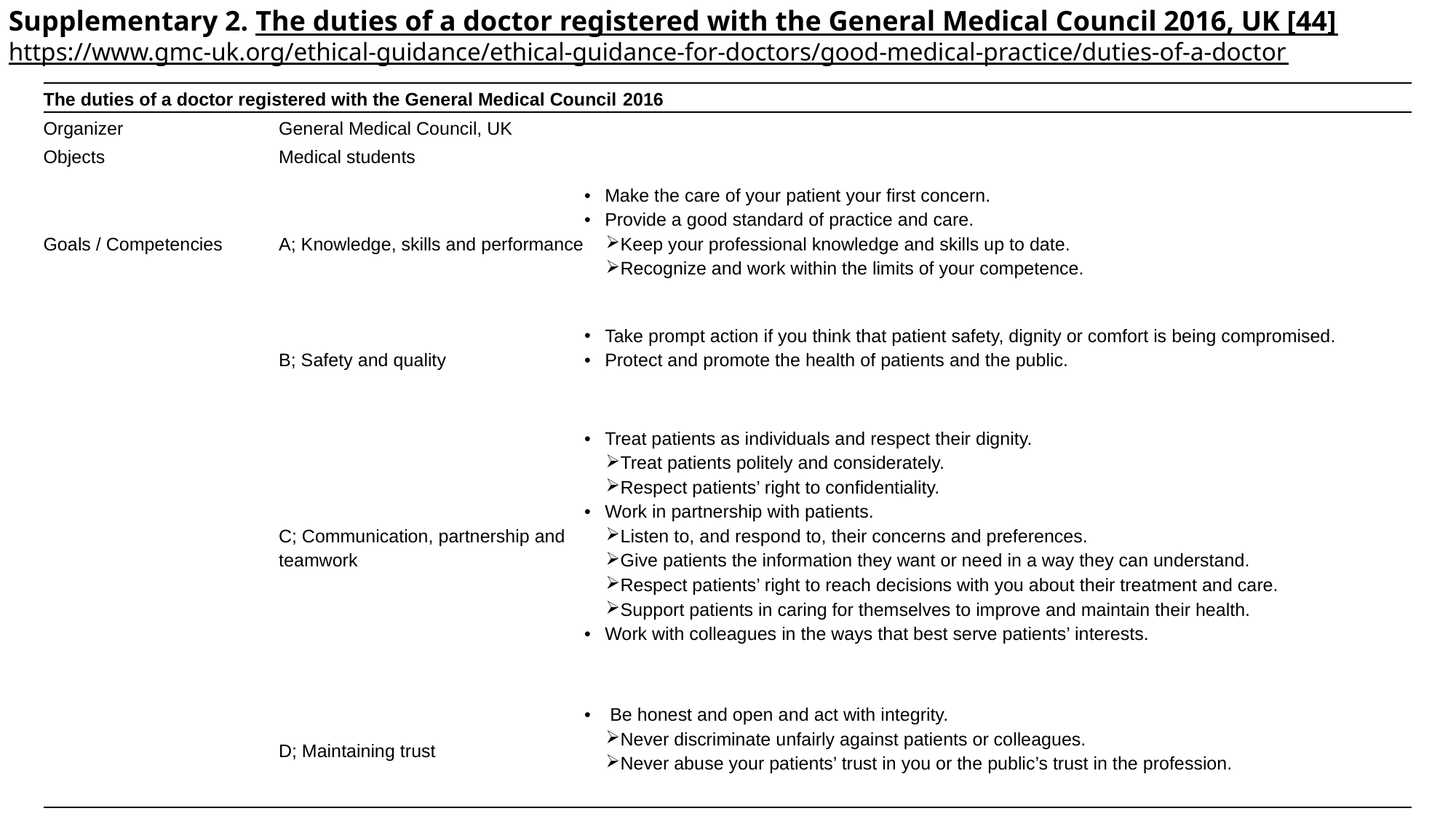

Supplementary 2. The duties of a doctor registered with the General Medical Council 2016, UK [44]
https://www.gmc-uk.org/ethical-guidance/ethical-guidance-for-doctors/good-medical-practice/duties-of-a-doctor
| The duties of a doctor registered with the General Medical Council 2016 | | |
| --- | --- | --- |
| Organizer | General Medical Council, UK | |
| Objects | Medical students | |
| Goals / Competencies | A; Knowledge, skills and performance | Make the care of your patient your first concern. Provide a good standard of practice and care. Keep your professional knowledge and skills up to date. Recognize and work within the limits of your competence. |
| | B; Safety and quality | Take prompt action if you think that patient safety, dignity or comfort is being compromised. Protect and promote the health of patients and the public. |
| | C; Communication, partnership and teamwork | Treat patients as individuals and respect their dignity. Treat patients politely and considerately. Respect patients’ right to confidentiality. Work in partnership with patients. Listen to, and respond to, their concerns and preferences. Give patients the information they want or need in a way they can understand. Respect patients’ right to reach decisions with you about their treatment and care. Support patients in caring for themselves to improve and maintain their health. Work with colleagues in the ways that best serve patients’ interests. |
| | D; Maintaining trust | Be honest and open and act with integrity. Never discriminate unfairly against patients or colleagues. Never abuse your patients’ trust in you or the public’s trust in the profession. |

## Slide 3
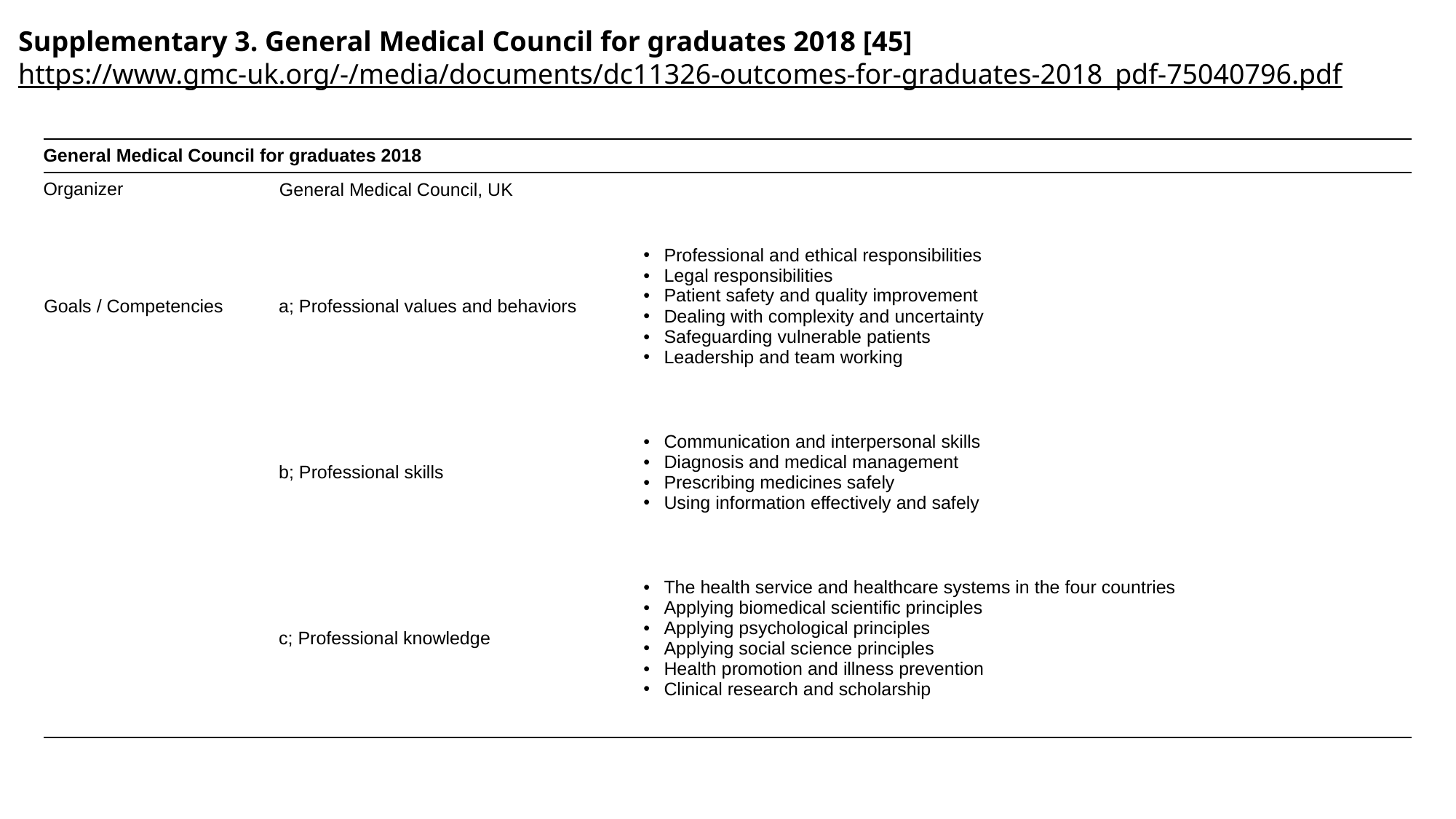

Supplementary 3. General Medical Council for graduates 2018 [45]
https://www.gmc-uk.org/-/media/documents/dc11326-outcomes-for-graduates-2018_pdf-75040796.pdf
| General Medical Council for graduates 2018 | | |
| --- | --- | --- |
| Organizer | General Medical Council, UK | |
| Goals / Competencies | a; Professional values and behaviors | Professional and ethical responsibilities Legal responsibilities Patient safety and quality improvement Dealing with complexity and uncertainty Safeguarding vulnerable patients Leadership and team working |
| | b; Professional skills | Communication and interpersonal skills Diagnosis and medical management Prescribing medicines safely Using information effectively and safely |
| | c; Professional knowledge | The health service and healthcare systems in the four countries Applying biomedical scientific principles Applying psychological principles Applying social science principles Health promotion and illness prevention Clinical research and scholarship |

## Slide 4
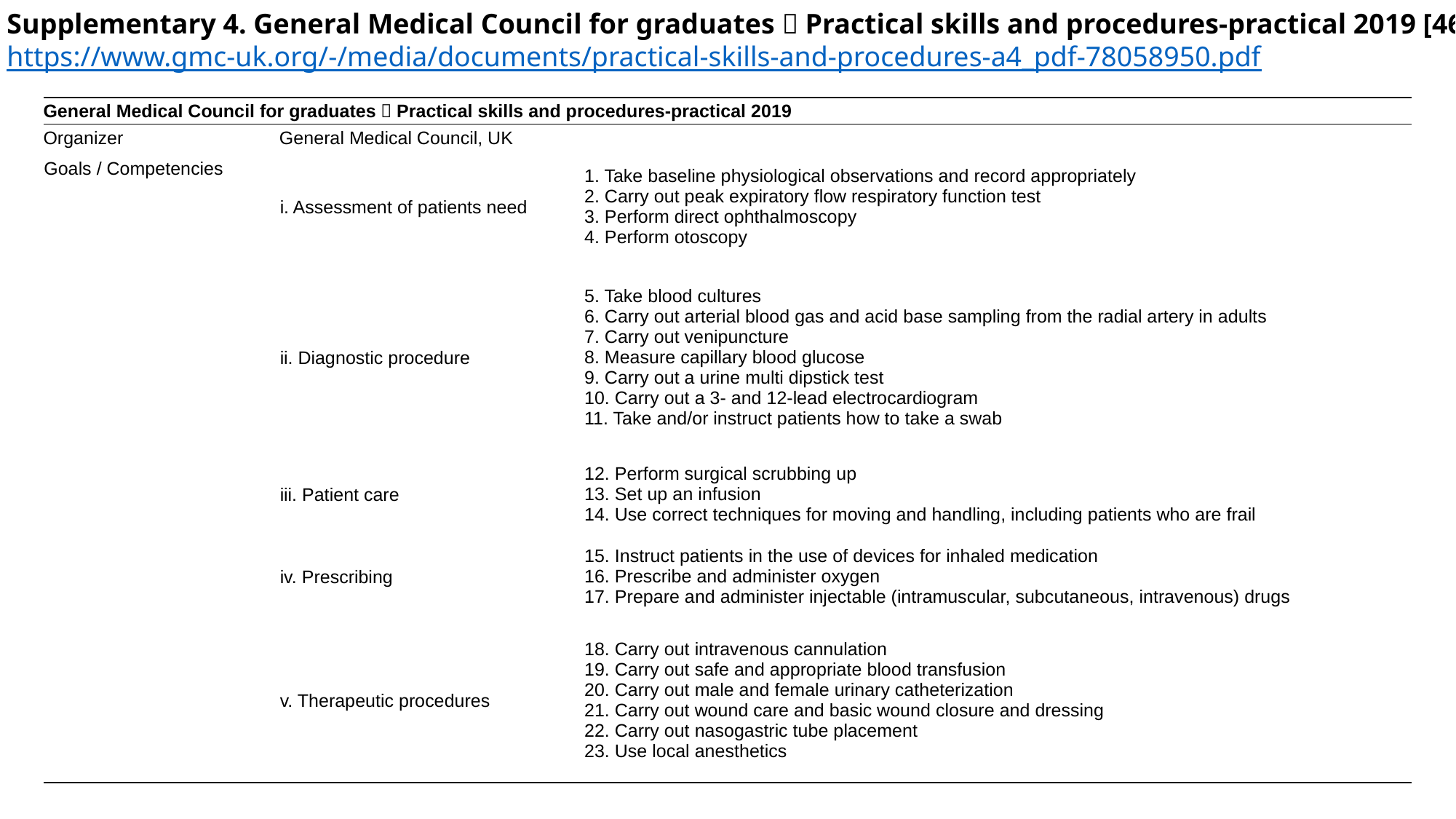

Supplementary 4. General Medical Council for graduates：Practical skills and procedures-practical 2019 [46]
https://www.gmc-uk.org/-/media/documents/practical-skills-and-procedures-a4_pdf-78058950.pdf
| General Medical Council for graduates：Practical skills and procedures-practical 2019 | | |
| --- | --- | --- |
| Organizer | General Medical Council, UK | |
| Goals / Competencies | i. Assessment of patients need | 1. Take baseline physiological observations and record appropriately 2. Carry out peak expiratory flow respiratory function test 3. Perform direct ophthalmoscopy 4. Perform otoscopy |
| | | |
| | | |
| | | |
| | ii. Diagnostic procedure | 5. Take blood cultures 6. Carry out arterial blood gas and acid base sampling from the radial artery in adults 7. Carry out venipuncture 8. Measure capillary blood glucose 9. Carry out a urine multi dipstick test 10. Carry out a 3- and 12-lead electrocardiogram 11. Take and/or instruct patients how to take a swab |
| | | |
| | | |
| | | |
| | | |
| | | |
| | | |
| | iii. Patient care | 12. Perform surgical scrubbing up 13. Set up an infusion 14. Use correct techniques for moving and handling, including patients who are frail |
| | | |
| | | |
| | iv. Prescribing | 15. Instruct patients in the use of devices for inhaled medication 16. Prescribe and administer oxygen 17. Prepare and administer injectable (intramuscular, subcutaneous, intravenous) drugs |
| | | |
| | | |
| | v. Therapeutic procedures | 18. Carry out intravenous cannulation 19. Carry out safe and appropriate blood transfusion 20. Carry out male and female urinary catheterization 21. Carry out wound care and basic wound closure and dressing 22. Carry out nasogastric tube placement 23. Use local anesthetics |
| | | |
| | | |
| | | |
| | | |
| | | |

## Slide 5
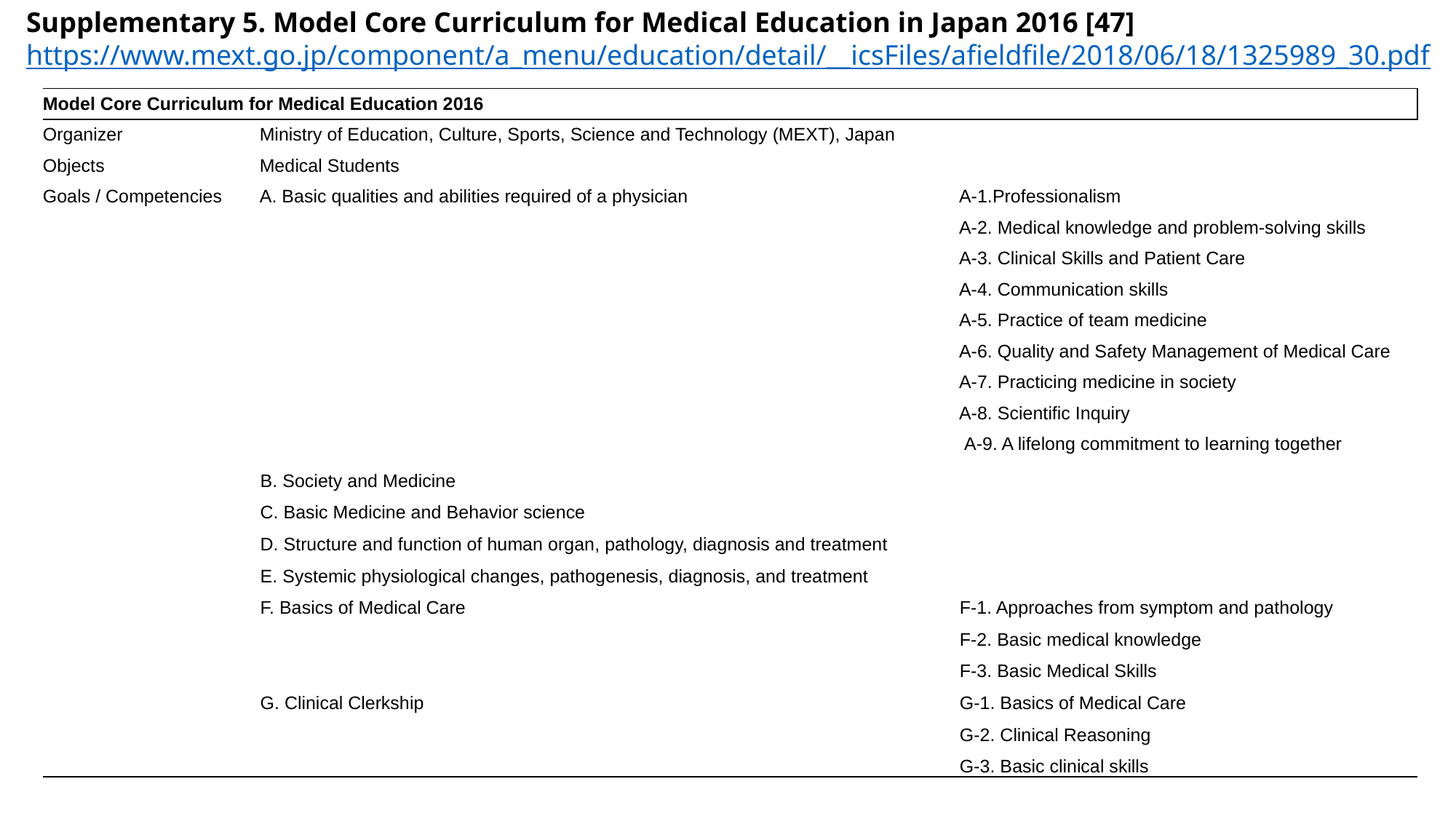

Supplementary 5. Model Core Curriculum for Medical Education in Japan 2016 [47]
https://www.mext.go.jp/component/a_menu/education/detail/__icsFiles/afieldfile/2018/06/18/1325989_30.pdf
| Model Core Curriculum for Medical Education 2016 | | |
| --- | --- | --- |
| Organizer | Ministry of Education, Culture, Sports, Science and Technology (MEXT), Japan | |
| Objects | Medical Students | |
| Goals / Competencies | A. Basic qualities and abilities required of a physician | A-1.Professionalism |
| | | A-2. Medical knowledge and problem-solving skills |
| | | A-3. Clinical Skills and Patient Care |
| | | A-4. Communication skills |
| | | A-5. Practice of team medicine |
| | | A-6. Quality and Safety Management of Medical Care |
| | | A-7. Practicing medicine in society |
| | | A-8. Scientific Inquiry |
| | | A-9. A lifelong commitment to learning together |
| | B. Society and Medicine | |
| | C. Basic Medicine and Behavior science | |
| | D. Structure and function of human organ, pathology, diagnosis and treatment | |
| | E. Systemic physiological changes, pathogenesis, diagnosis, and treatment | |
| | F. Basics of Medical Care | F-1. Approaches from symptom and pathology |
| | | F-2. Basic medical knowledge |
| | | F-3. Basic Medical Skills |
| | G. Clinical Clerkship | G-1. Basics of Medical Care |
| | | G-2. Clinical Reasoning |
| | | G-3. Basic clinical skills |

## Slide 6
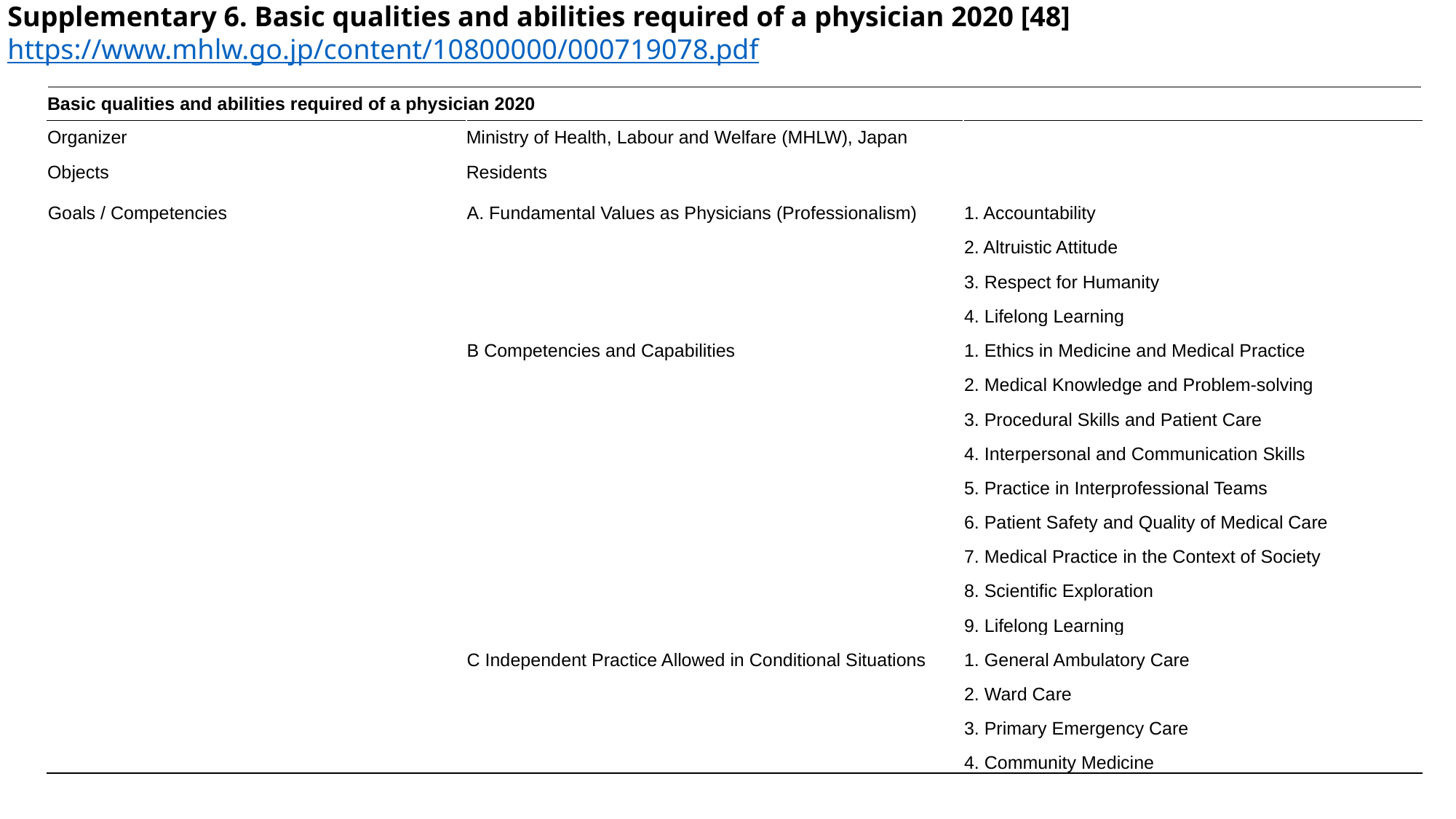

Supplementary 6. Basic qualities and abilities required of a physician 2020 [48]
https://www.mhlw.go.jp/content/10800000/000719078.pdf
| Basic qualities and abilities required of a physician 2020 | | |
| --- | --- | --- |
| Organizer | Ministry of Health, Labour and Welfare (MHLW), Japan | |
| Objects | Residents | |
| Goals / Competencies | A. Fundamental Values as Physicians (Professionalism) | 1. Accountability |
| | | 2. Altruistic Attitude |
| | | 3. Respect for Humanity |
| | | 4. Lifelong Learning |
| | B Competencies and Capabilities | 1. Ethics in Medicine and Medical Practice |
| | | 2. Medical Knowledge and Problem-solving |
| | | 3. Procedural Skills and Patient Care |
| | | 4. Interpersonal and Communication Skills |
| | | 5. Practice in Interprofessional Teams |
| | | 6. Patient Safety and Quality of Medical Care |
| | | 7. Medical Practice in the Context of Society |
| | | 8. Scientific Exploration |
| | | 9. Lifelong Learning |
| | C Independent Practice Allowed in Conditional Situations | 1. General Ambulatory Care |
| | | 2. Ward Care |
| | | 3. Primary Emergency Care |
| | | 4. Community Medicine |

## Slide 7
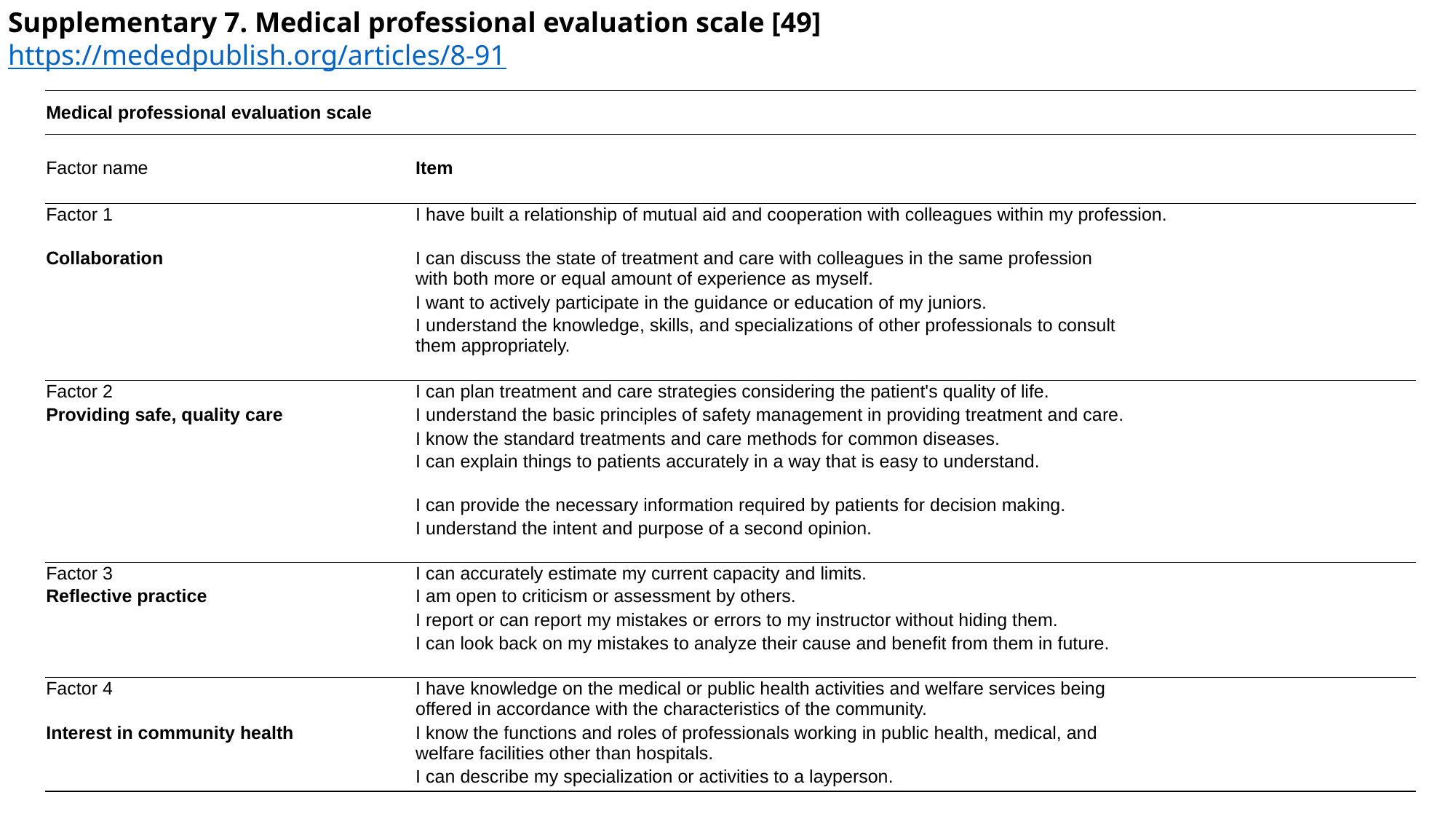

Supplementary 7. Medical professional evaluation scale [49]
https://mededpublish.org/articles/8-91
| Medical professional evaluation scale | | | | | | | | |
| --- | --- | --- | --- | --- | --- | --- | --- | --- |
| | | | | | | | | |
| Factor name | Item | | | | | | | |
| | | | | | | | | |
| Factor 1 | I have built a relationship of mutual aid and cooperation with colleagues within my profession. | | | | | | | |
| Collaboration | I can discuss the state of treatment and care with colleagues in the same profession with both more or equal amount of experience as myself. | | | | | | | |
| | I want to actively participate in the guidance or education of my juniors. | | | | | | | |
| | I understand the knowledge, skills, and specializations of other professionals to consult them appropriately. | | | | | | | |
| | | | | | | | | |
| Factor 2 | I can plan treatment and care strategies considering the patient's quality of life. | | | | | | | |
| Providing safe, quality care | I understand the basic principles of safety management in providing treatment and care. | | | | | | | |
| | I know the standard treatments and care methods for common diseases. | | | | | | | |
| | I can explain things to patients accurately in a way that is easy to understand. | | | | | | | |
| | I can provide the necessary information required by patients for decision making. | | | | | | | |
| | I understand the intent and purpose of a second opinion. | | | | | | | |
| | | | | | | | | |
| Factor 3 | I can accurately estimate my current capacity and limits. | | | | | | | |
| Reflective practice | I am open to criticism or assessment by others. | | | | | | | |
| | I report or can report my mistakes or errors to my instructor without hiding them. | | | | | | | |
| | I can look back on my mistakes to analyze their cause and benefit from them in future. | | | | | | | |
| | | | | | | | | |
| Factor 4 | I have knowledge on the medical or public health activities and welfare services being offered in accordance with the characteristics of the community. | | | | | | | |
| Interest in community health | I know the functions and roles of professionals working in public health, medical, and welfare facilities other than hospitals. | | | | | | | |
| | I can describe my specialization or activities to a layperson. | | | | | | | |
